# Chromosomal evolution of the *PKD1 *gene family in primates

**DOI:** 10.1186/1471-2148-8-263

**Published:** 2008-09-26

**Authors:** Stefan Kirsch, Juanjo Pasantes, Andreas Wolf, Nadia Bogdanova, Claudia Münch, Petra Pennekamp, Michael Krawczak, Bernd Dworniczak, Werner Schempp

**Affiliations:** 1 Institut für Humangenetik und Anthropologie, Universität Freiburg, Breisacher Str. 33, 79106 Freiburg, Germany; 2 Institut für Medizinische Informatik und Statistik, Universität Kiel, Brunswiker Str. 10, 24105 Kiel, Germany; 3 Institut für Humangenetik, Universität Münster, Vesaliusweg 12-14, 48129 Münster, Germany; 4 Department of Biochemistry, Genetics & Immunology, University of Vigo, E-36200 Vigo, Spain

## Abstract

**Background:**

The autosomal dominant polycystic kidney disease (ADPKD) is mostly caused by mutations in the *PKD1 *(polycystic kidney disease 1) gene located in 16p13.3. Moreover, there are six pseudogenes of *PKD1 *that are located proximal to the master gene in 16p13.1. In contrast, no pseudogene could be detected in the mouse genome, only a single copy gene on chromosome 17. The question arises how the human situation originated phylogenetically. To address this question we applied comparative FISH-mapping of a human *PKD1*-containing genomic BAC clone and a *PKD1*-cDNA clone to chromosomes of a variety of primate species and the dog as a non-primate outgroup species.

**Results:**

Comparative FISH with the *PKD1*-cDNA clone clearly shows that in all primate species studied distinct single signals map in subtelomeric chromosomal positions orthologous to the short arm of human chromosome 16 harbouring the master *PKD1 *gene. Only in human and African great apes, but not in orangutan, FISH with both BAC and cDNA clones reveals additional signal clusters located proximal of and clearly separated from the *PKD1 *master genes indicating the chromosomal position of *PKD1 *pseudogenes in 16p of these species, respectively. Indeed, this is in accordance with sequencing data in human, chimpanzee and orangutan. Apart from the master *PKD1 *gene, six pseudogenes are identified in both, human and chimpanzee, while only a single-copy gene is present in the whole-genome sequence of orangutan. The phylogenetic reconstruction of the *PKD1*-tree reveals that all human pseudogenes are closely related to the human *PKD1 *gene, and all chimpanzee pseudogenes are closely related to the chimpanzee *PKD1 *gene. However, our statistical analyses provide strong indication that gene conversion events may have occurred within the *PKD1 *family members of human and chimpanzee, respectively.

**Conclusion:**

*PKD1 *must have undergone amplification very recently in hominid evolution. Duplicative transposition of the *PKD1 *gene and further amplification and evolution of the *PKD1 *pseudogenes may have arisen in a common ancestor of *Homo*, *Pan *and *Gorilla *~8 MYA. Reticulate evolutionary processes such as gene conversion and non-allelic homologous recombination (NAHR) may have resulted in concerted evolution of *PKD1 *family members in human and chimpanzee and, thus, simulate an independent evolution of the *PKD1 *pseudogenes from their master *PKD1 *genes in human and chimpanzee.

## Background

Autosomal dominant polycystic kidney disease (ADPKD) is a late onset systemic disorder characterised by the progressive development of multiple fluid filled cysts in the kidney, ultimately leading to renal failure [1]. Peters and Sandkuijl [2] estimated that in affected individuals of European descent approximately 85% of ADPKD is due to mutations in the gene *PKD1 *(polycystic kidney disease 1), located on chromosome 16p13.3. About 50 kb of the *PKD1 *region in 16p13.3 is inserted and reiterated in several copies in 16p13.1, comprising six pseudogenes [3-5]. Only 3.5 kb of the *PKD1 *transcript, located at the 3'end of the gene, is unique to *PKD1 *[6]. In contrast to the human situation there is only one *Pkd1 *gene on mouse chromosome 17 and no further pseudogenes could be detected [7]. The question arose how the human situation originated phylogenetically. Besides single-base-pair mutations, sequence duplications and chromosomal rearrangements are the primary forces by which any genome evolves over time [8,9]. Various models of genomic duplications, e.g. the duplicative transposition or the endoduplication, have been documented. The duplicative transposition of a genomic block of material (1–100 kb) leads to segmental duplications within a chromosome/genome [10], which are also known as low copy repeat sequences, that mediate recurrent chromosomal structural rearrangements [4]. These segmental duplications are often harbouring a part of a gene containing intron and exon structures, which leads to the accumulation of unprocessed pseudogenes. Such duplications appear to have arisen in very recent evolutionary time (during the last 35 Myr), as judged by the high sequence identity (90–100%) seen both in introns and exons [4,9]. Interestingly, human chromosome 16 is one of the most enriched chromosomes for segmental duplications. They are particularly clustered along the p arm of the chromosome [5]. In contrast to duplicative transpositions, endoduplications originate from tandem duplication events of local chromosomal regions mediated by unequal crossover. In the case of *PKD1 *both models of genomic duplications can be implicated. First, the duplicative transposition as a hallmark for the separation of the pseudogenes from the *PKD1 *gene and second, the endoduplication among the pseudogenes as some of them are located next to each other in the same orientation, indicating a tandem duplication event [3,11].

Fluorescence *in situ *hybridization (FISH) is the main physical mapping tool to identify chromosomal rearrangements among closely related species [9]. Therefore we applied comparative FISH-mapping of a human *PKD1*-containing BAC clone and a *PKD1*-cDNA clone to chromosomes of human, great apes, gibbon, Old World and New World monkeys, lemurs and the dog as a non-primate outgroup species. Here we report the localization of the *PKD1 *gene and its pseudogenes. Moreover, with the advent of whole-genome sequencing, a highly accurate human genome sequence [4] and draft sequences of the chimpanzee [12], the orangutan [13], the rhesus macaque [14] and the common marmoset [15] genome have been generated. This offers the possibility for phylogenetic and comparative analyses of human and chimpanzee sequences of *PKD1 *genes and pseudogenes taking the rhesus macaque as an outgroup.

## Methods

### Tissue samples

Blood samples of chimpanzee (*Pan troglodytes*, PTR), pygmy chimpanzee *(Pan paniscus*, PPA), lowland gorilla (*Gorilla gorilla gorilla*, GGO), Sumatran orangutan (*Pongo pygmaeus abelii*, PPYsu), and Bornean orangutan (*Pongo pygmaeus pygmaeus*, PPYbo) were obtained from the Zoologisch-Botanischer Garten Wilhelma in Stuttgart, Germany, the Zoologischer Garten Berlin, Germany, and the Zoologischer Garten Leipzig, Germany. Blood samples of the lar gibbon (*Hylobates lar*, HLA), the proboscis monkey (*Nasalis larvatus*, NLA), and the black-handed spider monkey (*Ateles geoffroyi*, AGE) were also obtained from the Zoologisch-Botanischer Garten Wilhelma in Stuttgart, Germany. Blood probes of the Mueller's or grey gibbon (*Hylobates muelleri*, HMU), the rhesus macaque (*Macaca mulatta*, MMU), the pig-tailed macaque (*Macaca nemestrina*, MNE), and the ring-tailed lemur (*Lemur catta*, LCA) were received from the Zoologischer Garten Münster, Germany, the Zoologischer Garten Antwerpen, Belgium, and the Deutsches Primatenzentrum in Göttingen, Germany. From Prof. Dr. Y. Rumpler in Strasbourgh, France, we received blood samples from the ring-tailed lemur (*Lemur catta*, LCA), and the brown lemur (*Eulemur fulvus*, EFU). Blood probes from the domestic dog (*Canis lupus f. familiaris*, CFA) we got from Dr. R. Stanyon in Firenze, Italy.

### Chromosome preparation

Standard chromosome preparation methods were applied to peripheral lymphocyte cultures of human, primates and the domestic dog [16].

### Fluorescence in situ hybridization (FISH)

Prior to FISH, the slides were treated with RNase followed by pepsin digestion as described in [17]. FISH followed essentially the methods already described in [16]. Chromosome *in situ *suppression was applied to the probes BAC RP11-304L19 (Ensembl release 47: [AC009065]; Chr 16: 2,081,103 – 2,267,413) which harbours the complete *PKD1 *gene and several additional genes. The BAC was originally purchased from Research Genetics, Inc., (Huntsville, Ala, USA now Invitrogen GmbH, Germany) and a 6.5 kb long partial *PKD1*-cDNA clone covering the exons 15–31 of the *PKD1 *gene (generously provided by Peter Harris; Division of Nephrology and Hypertension, Mayo Clinic College of Medicine, Rochester, MN, USA). In our FISH-experiments BAC clone RP11-304L19 was referred to as "BAC", the *PKD1*-cDNA clone as "cDNA". After FISH the slides were counterstained with DAPI (0.14 μg/ml) and mounted in Vectashield (Vector Laboratories).

### Fluorescence microscopy and imaging

Preparations were evaluated using a Zeiss Axiophot epifluorescence microscope equipped with single-band pass filters for excitation of red, green and blue (Chroma Technologies, Brattleboro, VT). During exposures, only excitation filters were changed allowing for pixel-shift-free image recording. Images of high magnification and resolution were obtained using a black-and-white CCD camera (Photometrics Kodak KAF 1400; Tucson, AZ) connected to the Axiophot. Camera control and digital image acquisition involved the use of an Apple Macintosh Quadra 950 computer.

### Sequence conservation analyses

Sequences orthologous to human *PKD1 *intron 30 were identified in the whole genome assemblies of the rat (rn4), mouse (NCBI Build 37; mm9), cat (felCat3), dog (canFam2), horse (equCab1), and opossum (monDom4) by using human intron 30 anchored with flanking exons as a query sequence. All mammalian sequences were extracted and multiple sequence alignments carried out using CLUSTALW 2.0 [18]. Pairwise identities for intron 30 were calculated directly from the CLUSTALW alignments.

### Phylogenetic analyses

Human sequences paralogous to the human *PKD1 *master gene genomic locus (NC_000016: bp 2,078,712 – 2,125,900) were obtained from GenBank as follows: *PKD1*P1: NG_002797; *PKD1*P2: NG_002795; *PKD1*P3: NG_002796; *PKD1*P4: NG_002800; *PKD1*P5: NG_002798; *PKD1*P6: NG_002799. Phylogenetic analysis was focused on the largest intron (> 1.5 kb) common for all gene loci (intron 30). Subsequently, primate sequences orthologous and paralogous to the human intron 30 sequence were identified in the whole genome sequence assemblies of the chimpanzee (panTro2), orangutan (ponAbe2), and rhesus macaque (rheMac2). Sequences were extracted, multiple sequence alignments generated using CLUSTALW 2.0 [18] and gap-containing positions removed. Pairwise identities of all sequences were based on the CLUSTALW alignments. MODELTEST [19] was used to select the model of nucleotide substitution best fitting the sequences. The nucleotide sequence phylogeny was built using an ML model for nucleotide data with the parameters defined by MODELTEST (PAUP* 4.0; [20]). The *PKD1 *intron 30 topology was initially assessed using parsimony and distance-related nonparametric bootstrapping. No topological differences were noted for the nucleotide phylogenies.

The topology of the phylogenetic tree was used to match pairs of human and chimpanzee pseudogene copies according to their comparable positions within the phylogram. As a reference, the pair of human and chimpanzee master gene sequences was used. Subsequently, seven independent multiple alignments were generated by CLUSTALW 2.0 [18], gap-containing positions removed and a phylogenetic tree created using Dnaml 3.5 c [21]. Branch lengths including confidence limits and p-values (likelihood ratio tests) are documented in Table 1. Strong indication for the correctness of the branch length is noted when two gene/pseudogene copies had a p-value that was less than 0.05 for five different types of statistical tests.

**Table 1:** Dnaml analysis of matched pairs of human/chimpanzee genes/pseudogenes

Matched pairs	Branching point to	Branch length	Approx. Confidence limits
HSA*PKD1*/PTR*PKD1*	HSA*PKD1*	0.01290	0.00553 0.02032 **
	PTR*PKD1*	0.01365	0.00603 0.02124 **
HSA*PKD1*P1/PTR*PKD1*P1	HSA*PKD1*P1	0.01091	0.00462 0.01719 **
	PTR*PKD1*P1	0.01121	0.00488 0.01753 **
HSA*PKD1*P2/PTR*PKD1*P2	HSA*PKD1*P2	0.00865	0.00297 0.01430 **
	PTR*PKD1*P2	0.01171	0.00526 0.01820 **
HSA*PKD1*P3/PTR*PKD1*P6	HSA*PKD1*P3	0.00986	0.00333 0.01641 **
	PTR*PKD1*P6	0.03514	0.02301 0.04728 **
HSAPKD1P4/PTR*PKD1*P4	HSA*PKD1*P4	0.00794	0.00241 0.01347 **
	PTR*PKD1*P4	0.01507	0.00769 0.02250 **
HSA*PKD1*P5/PTR*PKD1*P5	HSA*PKD1*P5	0.00610	0.00106 0.01102 **
	PTR*PKD1*P5	0.01390	0.00653 0.02132 **
HSA*PKD1*P6/PTR*PKD1*P3	HSA*PKD1*P6	0.01267	0.00597 0.01937 **
	PTR*PKD1*P3	0.01229	0.00572 0.01887 **

** = significantly positive, P < 0.01

Human and chimpanzee *PKD1 *intron 30 sequences were further analyzed using GENECONV 1.81 [22]. Fragments shared by a pair of DNA sequences in a multiple alignment presenting more consecutive identical polymorphic sites in common than expected by chance are identified. Evidence for gene conversion is indicated when a fragment had a p-value less than 0.05 after multiple correction of the p-values.

## Results

The chromosomal location of *PKD1 *sequences was defined by applying one- and two-colour fluorescence *in situ *hybridisation (FISH) of two human-derived probes, the *PKD1*-containing BAC clone RP11-304L19 and the *PKD1*-cDNA clone HG 31918N2, to prometaphase or metaphase chromosomes of human, great apes, gibbon, Old World and New World monkeys, lemurs and the dog as a non-primate outgroup species. During our search for *PKD1 *sequences in primates and one representative of the carnivores, the orthologous regions to the human short arm chromosome 16 could be identified with the assistance of several published chromosomal painting papers. Presenting our comparative FISH-results in human and great apes we applied the system of orthologous numbering of human and great ape chromosomes [23].

### Comparative mapping in human and great apes

**Figure 1:**
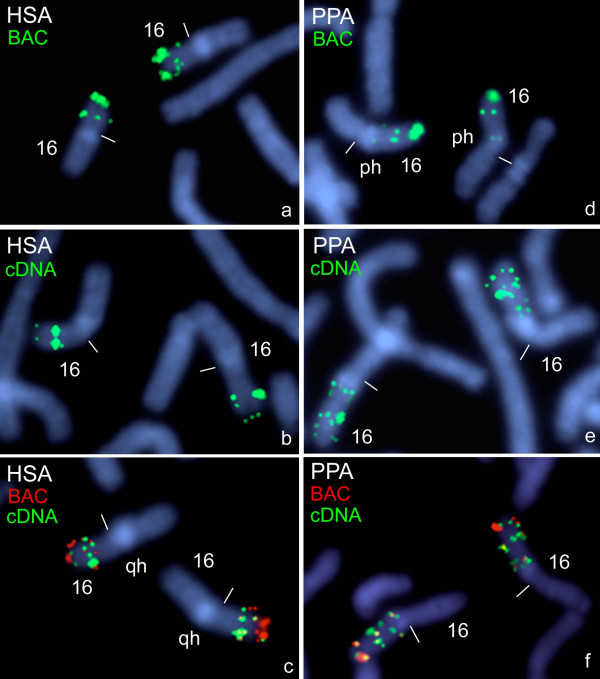
**Hybridization of *PKD1 *probes on human and bonobo chromosomes**. One- and two-colour FISH of human *PKD1 *BAC clone RP11-304L19 (BAC) and *PKD1 *cDNA clone (cDNA) to prometaphase chromosome 16 of human (HSA) **(a-c**) and to prometaphase chromosome 16 of the bonobo (PPA) **(d-f)**. The colours of the inserted clone names correspond to those of the fluorescence signals (biotin-FITC: green; digoxigenin-TRITC: red; yellow: overlapping green and red signals) on the DAPI-counterstained plates. "qh" and "ph" mean long arm and short arm heterochromatin. Centromeres are marked by small bars.

FISH with the BAC clone on human chromosomes revealed the expected prominent *PKD1*- signal distal in 16p13.3, as well as a signal cluster in 16p13.1, where the pseudogenes are localized (Fig. 1a+c). Hybridizations with the cDNA clone confirmed the localizations found with the BAC clone, but showed weaker *PKD1*-signals in 16p13.3 (Fig. 1b+c).

For both chimpanzee and bonobo chromosomes 16 identical FISH-results were obtained. Three clearly separated *PKD1*-signals could be detected with the BAC clone, showing a strong *PKD1*-signal in distal 16p15, weaker signals in proximal 16p15 and a third distinct signal in 16p13, distal to the short arm heterochromatin (Fig. 1d+f). Detection with the cDNA clone could confirm these three localizations, but showed weaker *PKD1*-signals in distal 16p15 (Fig. 1e+f). According to our results the DAPI-bright heterochromatin of both the chimpanzee and the bonobo chromosome 16 maps to the proximal short arm on the side of the *PKD1*-signals, but not to the proximal long arm as presented in the ideogrammatic drawing in ISCN [24]. Indeed, this pericentric inversion bringing the DAPI-bright heterochromatin to the proximal short arm of chimpanzee chromosome 16 was recently established by means of molecular breakpoint analysis [25].

**Figure 2:**
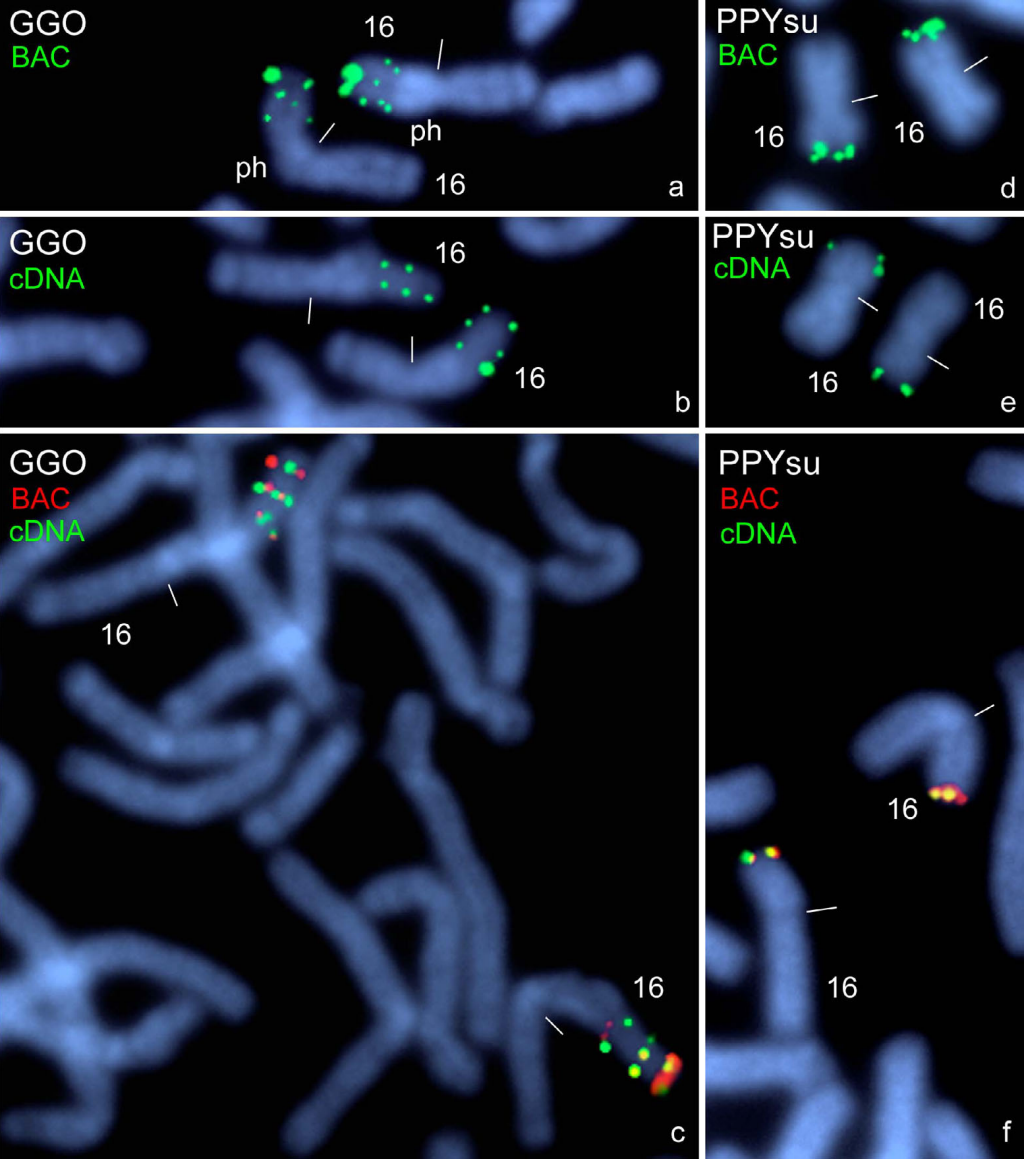
**Hybridization of *PKD1 *probes on gorilla and orangutan chromosomes**. One- and two-colour FISH of human BAC clone RP11-304L19 (BAC) and *PKD1*-cDNA clone (cDNA) to meta- and prometaphase chromosome 16 of the gorilla (GGO) **(a-c) **and to meta- and prometaphase chromosome 16 of the Sumatran orangutan (PPYsu) **(d-f)**. The colours of the inserted clone names correspond to those of the fluorescence signals (see Fig.1) on the DAPI-counterstained plates. "ph" stands for short arm heterochromatin. Centromeres are marked by small bars.

Likewise, in gorilla with the BAC clone three clearly separated *PKD1*-signals were detected in the short arm of chromosome 16. A strong signal was detected in distal 16p15, and two weaker signals in proximal 16p15 and in 16p13, respectively (Fig. 2a+c). Hybridizations with the cDNA clone revealed the same *PKD1*-signal pattern but with equal signal intensities for all three locations (Fig. 2b+c).

A different situation is given for the chromosome 16 of the orangutan subspecies from Sumatra. Our FISH results obtained with the BAC clone revealed strong *PKD1*-signals in distal 16p14 (Fig. 2d+f). Hybridizations with the cDNA clone confirmed this result, however, weaker *PKD1*-signals were detected (Fig. 2e+f). The same results we achieved for the Bornean orangutan (data not shown).

### Comparative mapping in gibbons and Old World monkeys

**Figure 3:**
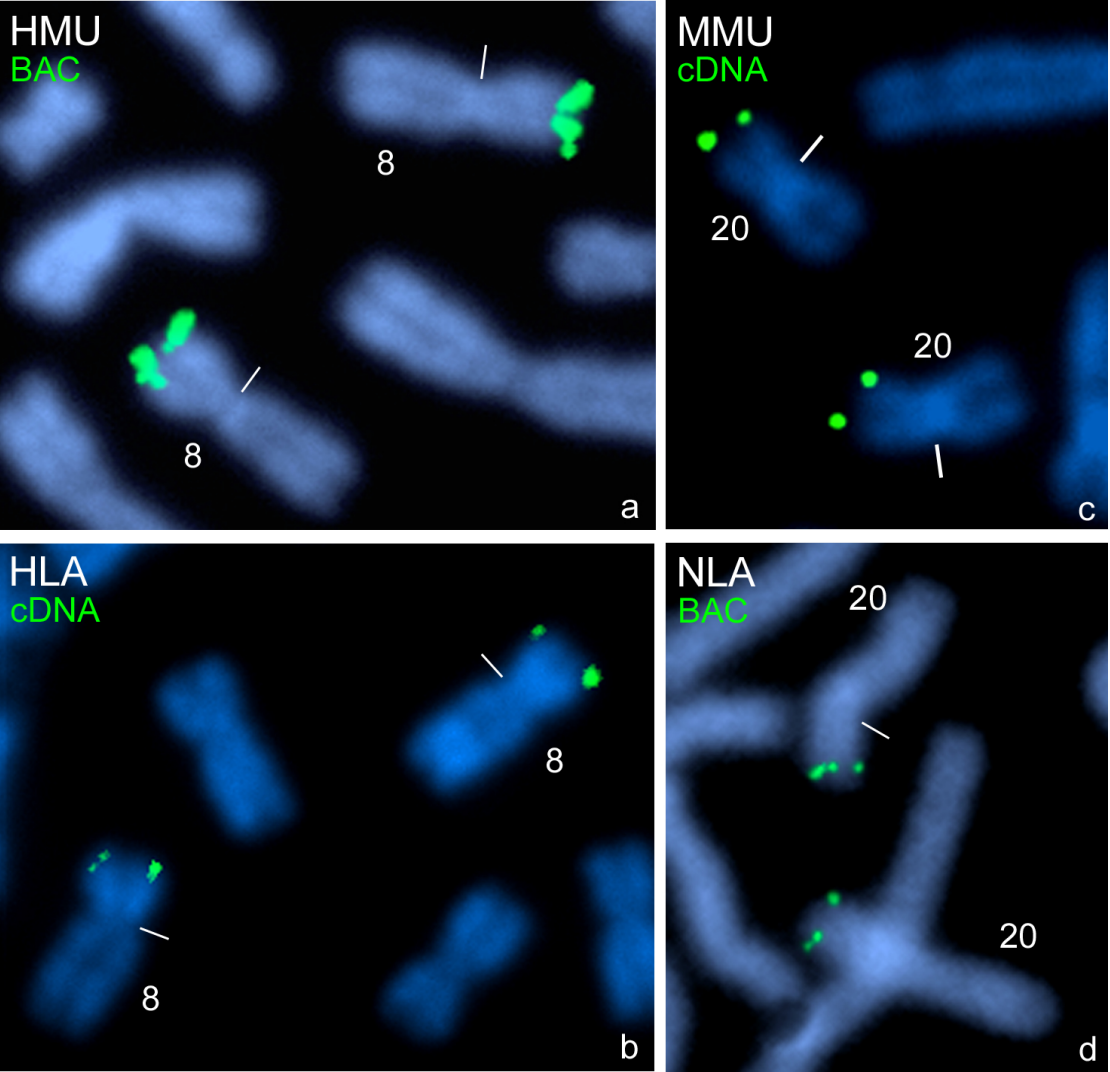
**Hybridization of *PKD1 *probes on gibbon and Old World monkey chromosomes**. FISH of human *PKD1 *BAC clone RP11-304L19 (BAC) to metaphase chromosome 8 of the Mueller's gibbon (HMU) **(a) **and of human *PKD1*cDNA clone (cDNA) to metaphase chromosome 8 of the white-handed gibbon (HLA) **(b)**. The identification of the distal short arm of chromosome 8 of both gibbon species as orthologous to human chromosome 16 is according to Jauch et al. [26] and Müller et al. [27]. Two-colour FISH of human BAC clone RP11-304L19 (BAC) and *PKD1*cDNA clone (cDNA) to metaphase chromosome 20 of the rhesus macaque (MMU) **(c)**. FISH of human BAC clone RP11-304L19 (BAC) to prometaphase chromosome 20 of the proboscis monkey (NLA) **(d)**. The identification of the chromosome 20 of the proboscis monkey as orthologous to human chromosome 16 is according to Bigoni et al. [31]. The colours of the inserted clone names correspond to those of the fluorescence signals (see Fig.1) on the DAPI-counterstained plates. Centromeres are marked by small bars.

Hybridizations with the BAC and cDNA clones on chromosomes of Mueller's gibbon (HMU), and lar gibbon (HLA) revealed strong *PKD1*-signals in distal 8p15 (Fig. 3a+b). The signals appear very strong and clustered after using the BAC as a probe (Fig. 3a, HMU) but weaker when the cDNA was used as a probe (Fig. 3b, HLA). This distal region in 8p15 was shown to be orthologous to human chromosome 16 [26,27]. FISH with BAC and cDNA clones on chromosomes of the rhesus macaque (MMU) and the pig-tailed macaque (MNE) revealed *PKD1*-signals in the distal short arm of chromosome 20 (Fig. 3c, MMU cDNA), the orthologous chromosome to human chromosome 16 [[28-30], see also http://www.biologia.uniba.it/macaque]. Using both the BAC and the cDNA clones on chromosomes of the proboscis monkey (NLA) *PKD1*-signals appeared again in the distal short arm of chromosome 20 (Fig. 3d), the orthologous chromosome to human chromosome 16 [31].

### Comparative mapping in a New World monkey, lemurs and the dog

**Figure 4:**
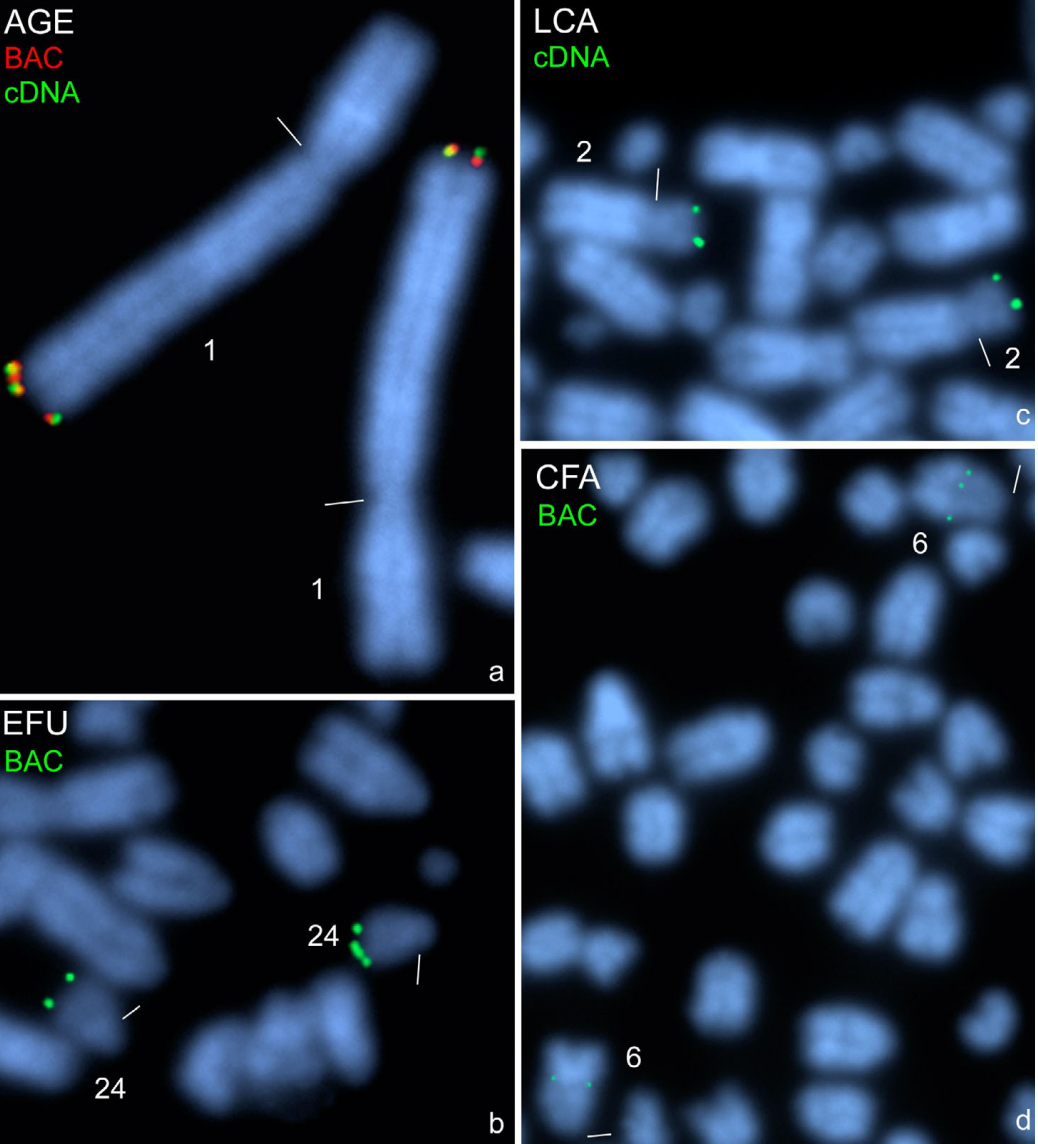
**Hybridization of *PKD1 *probes on New World monkey and lemur chromosomes**. Two-colour FISH of human BAC clone RP11-304L19 (BAC) and *PKD1*-cDNA clone (cDNA) to metaphase chromosome 1 of the black-handed spider monkey (AGE) **(a)**. The identification of the distal long arm of chromosome 1 of the spider monkey as orthologous to human chromosome 16 is according to Morescalchi et al. [32]. FISH of human BAC clone RP11-304L19 (BAC) to metaphase chromosome 24 of the brown lemur (EFU) **(b)**, to metaphase chromosome 2 of the ring-tailed lemur (LCA) **(c) **and to metaphase chromosome 6 of the domestic dog (CFA) **(d)**. The identification of the chromosome 24 of the brown lemur as orthologous to human chromosome 16 is according to Müller et al. [33], of the short arm of chromosome 2 of the ring-tailed lemur as orthologous to human 16p is according to Cardone et al.[34], and of chromosome 6 of the dog as orthologous to human 16p is according to Yang et al. [35]. The colours of the inserted clone names correspond to those of the fluorescence signals (see Fig.1) on the DAPI-counterstained plates. Centromeres are marked by small bars.

Performing FISH with BAC and cDNA clones on chromosomes of one representative of the New World monkeys, the black-handed spider monkey, we could unequivocally map distinct *PKD1*-signals distal in 1q26 (Fig. 4a, AGE), which could be defined as the orthologous region to human chromosome 16 [32]. Hybridizations with either the BAC or cDNA clone on lemur chromosomes revealed distinct *PKD1*-signals in the distal long arm of chromosome 24 of the brown lemur (Fig. 4b, EFU) and in the distal short arm of chromosome 2 of the ring-tailed lemur (Fig. 4c, LCA). Chromosome 24 of the brown lemur could be described as the orthologous chromosome to human chromosome 16 [33], and chromosome 2 of the ring-tailed lemur as orthologous to the short arm of human chromosome 16 [34]. FISH with the *PKD1 *BAC clone on chromosomes of the domestic dog revealed one very distinct *PKD1*-signal in the middle of the long arm of chromosome 6 (Fig. 4d, CFA), the orthologous chromosome to the short arm of human chromosome 16 [35].

### Comparative sequence and phylogenetic analyses

Based on the current genome reference sequence assemblies of human (NCBI build 36.3), chimpanzee (panTro2), orangutan (ponAbe2), rhesus macaque (rheMac2), and common marmoset (calJac1) we identified the master gene and 6 pseudogenes for *PKD1 *in human (HSA *PKD1*P1-P6) and chimpanzee (PTR *PKD1*P1 [NW_001226536], PTR *PKD1*P2 [NW_001225854], PTR *PKD1*P3 [NW_001225858], PTR *PKD1*P4 [NW_001226562], PTR *PKD1*P5 [NW_001225880], PTR *PKD1*P6 [NW_001226561]), respectively, while only a single-copy *PKD1 *gene was present in orangutan, rhesus macaque and common marmoset. Pairwise sequence comparisons of all pseudogene copies with the corresponding master gene revealed intron 30 as the longest stretch (> 1.5 kb) of non-coding DNA present in all genes/pseudogenes. It was previously shown that intron 45 of the *PKD1 *master gene shows a remarkable sequence conservation [36]. To assess the sequence conservation of intron 30, we performed multiple pairwise sequence comparisons of sequences orthologous to human intron 30 from six mammalian species (mouse, rat, cat, dog, human, opossum). Except for the mouse-rat pairwise alignment (83% nucleotide sequence identity), all other percent identities ranged from 45% to 56% indicating no sequence conservation. To analyze the phylogenetic relationships of master genes and pseudogenes, we compared all intron 30 sequences by carrying out a CLUSTALW alignment. Irrespective of the model of nucleotide substitution determined by MODELTEST [19] and the criteria used to analyze the sequence data in PAUP*4.0b [20], phylogenetic reconstruction of *PKD1 *using intron 30 always indicated that all human pseudogenes are closely related to the human *PKD1 *gene, and all chimpanzee pseudogenes are closely related to the chimpanzee *PKD1 *gene (Additional File 1). From an evolutionary point of view, and also with regard to our FISH results in human and great apes, it seems very unlikely that *PKD1 *pseudogenes originated independently in human and chimpanzee lineages. In addition, bearing in mind previous reports on pseudogene-mediated gene conversion to *PKD1 *[37-39], this prompted us to search for indications of concerted evolution in the *PKD1 *family members in human and chimpanzee.

### Indications for concerted evolution

In a first approach we made use of the phylogenetic tree of *PKD1 *intron 30 rooted on the rhesus macaque (Additional File 1). Based on the topology of this tree we matched seven pairs of orthologous *PKD1*-loci from human and chimpanzee (Table 1). Seven phylograms independently created with the *PKD1 *master genes of orangutan and rhesus macaque together with one pair of orthologous *PKD1 *loci from human and chimpanzee showed for all *PKD1 *loci, with the exception of human PKD1P3, almost identical branch lengths (expected number of nucleotide substitutions/site) of 0.020–0.027. This indicates that these pseudogenes may have originated before the human and chimpanzee lineages diverged.

Subsequently, we calculated the pairwise nucleotide sequence identities among all pseudogenes and the master genes of human, chimp, orangutan and rhesus macaque (Additional file 2).

In general, the human paralogous pseudogenes as well as the chimpanzee paralogous pseudogenes are more closely related among each other, respectively, than are the orthologous *PKD1 *master genes of both species. As reviewed in Chen et al. [40] this result is a clear indication that gene conversion may have occurred among *PKD1 *pseudogenes in human and chimpanzee, respectively.

In addition, the human and chimpanzee *PKD1 *intron 30 sequences were scanned for potential recombinant sequences by GENECONV analysis, which is a well-established method for detecting partial gene conversion [41]. In human, four DNA-fragments ranging in size from 522 bp to 637 bp were identified which are significantly more similar to each other than would be expected by chance, even after correction for multiple testing (Additional file 2). Thus, GENECONV indicates partial gene conversion between human *PKD1 *pseudogenes P2 and P3, P1 and P4, P3 and P4, as well as P1 and P2, respectively. Surprisingly, in the chimpanzee, a gene conversion between the *PKD1 *master gene and pseudogene 2 is indicated (Additional file 3).

Taken together, our three-pronged approach yielded substantial evidence for, at least, concerted evolution between *PKD1 *pseudogene copies in human.

## Discussion and conclusion

We applied comparative FISH-mapping with two human derived probes, the *PKD1*-containing genomic BAC clone RP11-304L19 and the *PKD1*-cDNA clone HG 31918N2, to localize *PKD1*-sequences on chromosomes of a variety of primates, including humans, and the dog as a non-primate outgroup-species.

**Figure 5:**
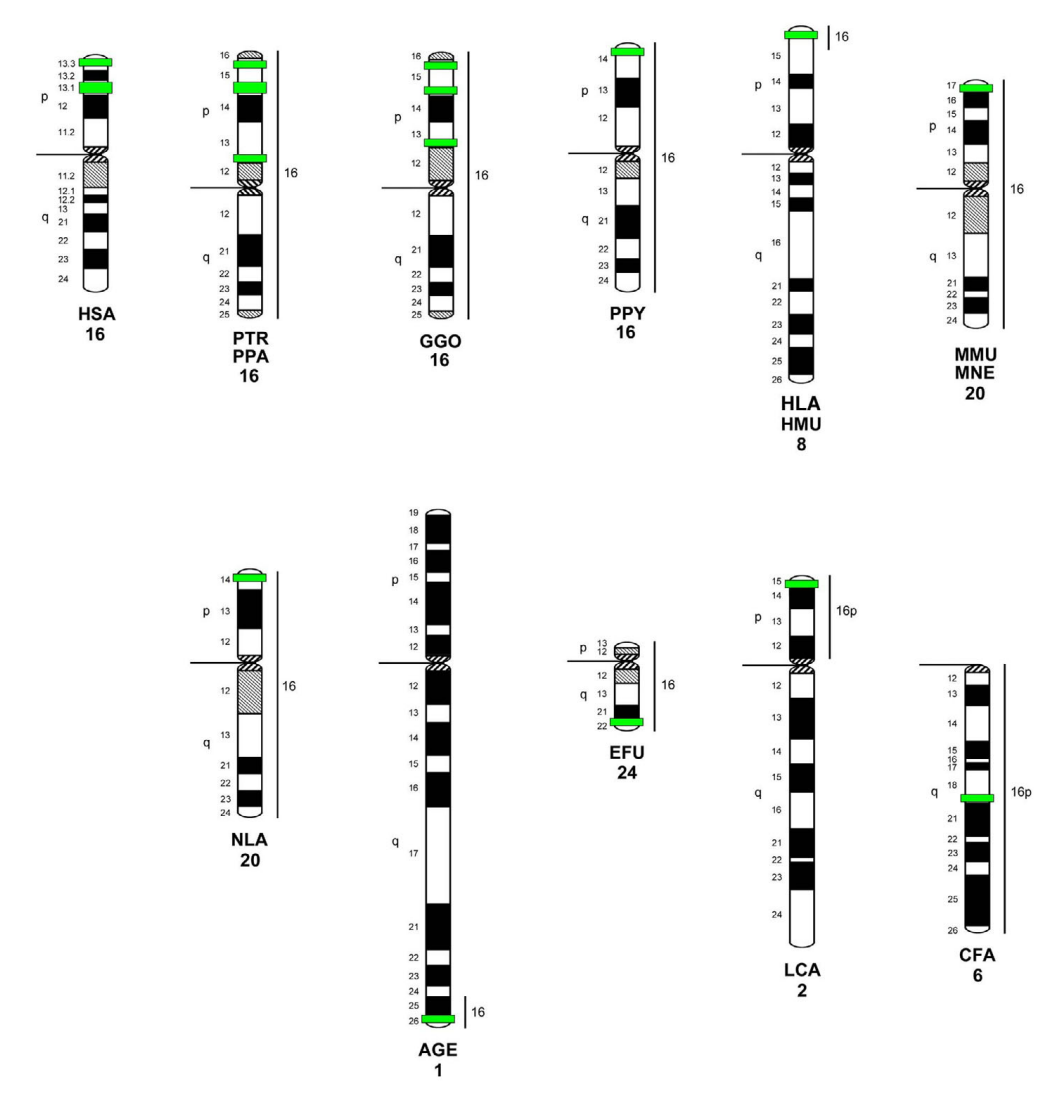
**Hybridization assignments of *PKD1 *on G-banded ideograms of primate chromosomes orthologous to human chromosome 16**. Together with the species names, the *PKD1*-bearing chromosomes are numbered below. *PKD1*-localizations are marked with green boxes. The homology to human chromosome 16 is marked through a vertical line on the right site of each chromosome ideogram. The G-banded ideograms for human and great apes are according to ISCN [24], the one for mueller's gibbon is according to Jauch et al. [26]. The ideogram for the rhesus macaque (MMU) and the pig-tailed macaque (MNE) is done after Wienberg et al. [30], as well as after own DAPI banding patterns, the one for the proboscis monkey (NLA) is done after our own DAPI banding patterns. The black-handed spider monkey's (AGE) ideogram is according to Morescalchi et al. [32], modified after our own DAPI banding patterns. The ideogram for the brown lemur (EFU) is done after Müller et al. [33], the one for the ring-tailed lemur (LCA) is done after Cardone et al. [34]. Both ideograms were also modified after our own DAPI banding patterns. The ideogram for the domestic dog (CFA) is according to Switonski et al. [47], and was adapted after Yang *et al*. [35].

In order to decide whether the *PKD1*-signals found in each species can be regarded as a single-copy signal or if duplications/amplifications must be taken into consideration, we compared our results with those obtained in human, in which the master *PKD1 *gene maps to 16p13.3 and six *PKD1 *pseudogenes map to 16p13.1 [4,5], and in the domestic dog, in which a single copy signal situation for the orthologous *PKD1 *gene is established [42]. As schematically summarized in Fig. 5, FISH with *PKD1 *clones in the human reveal a distinct and weaker signal in the distal short arm in 16p13.3 and a more proximal signal-cluster in 16p13.1, which is in accordance with the above cited human sequencing data. On the other hand, hybridization of the genomic BAC clone on chromosomes of the domestic dog detect a very distinct single signal confirming the results of Dackowski et al. [42], and allow us to map the orthologous *PKD1 *gene in 6q18 of the dog. As in the human, in chimpanzee, bonobo and gorilla a distinct and weaker signal can be detected in their distal short arms after FISH with *PKD1*-cDNA. However, in contrast to one proximal signal-cluster in human 16p13.1, two clearly separated signals appear in the proximal short arms of chimpanzee, bonobo and gorilla chromosomes 16. Summarizing our FISH results for *Homo*, *Pan *and *Gorilla*, the master *PKD1 *gene is located in the subtelomeric short arms of the orthologous chromosomes 16, respectively, while the chromosomal arrangement of the amplified pseudogenes may have occurred by further intrachromosomal rearrangements in a species-specific manner. This is not in contradiction to the work of Misceo et al. [43], having shown that the short arm of chromosome 16 is highly conserved in human and great apes. The BAC clones from human 16p they have used for comparative FISH (RP11-292B10, -291J20, -450G05, -360L15) neither interfere with the subtelomeric position of the *PKD1 *gene, nor with the map positions of the *PKD1 *pseudogenes, at least in the human.

Interestingly, in both orangutan subspecies from Sumatra and Borneo, and in all phylogenetically more distant species investigated only single *PKD1*-signals were detected at chromosomal sites orthologous to human chromosome 16. Thus, we may infer a single-copy *PKD1 *gene for all these species. Indeed, only a single *PKD1 *gene is present in the current genome sequence assembly of the orangutan [13], the rhesus macaque [14], and the common marmoset [15]. Due to the limited resolution of the FISH-technique we cannot exclude a duplication/amplification of *PKD1 *at the chromosomal signal sites of the other primates investigated.

In conclusion, with regard to our comparative FISH-data it seems very likely, that the original duplication of the *PKD1 *gene and further amplification and evolution of the *PKD1 *pseudogenes may have occurred in a common ancestor of *Homo*, *Pan *and *Gorilla *~8MYA.

Our combinatorial phylogenetic tree analysis using intron 30 from human and chimpanzee *PKD1*-loci, with orangutan and macaque as outgroup species, raises the impression of an independent evolution of the *PKD1 *pseudogenes from their master *PKD1 *genes in human and chimpanzee (Additional File 1). However, it should be noted that a complex genome architecture with all *PKD1 *pseudogenes being embedded in segmental duplications has been documented for the short arm of human [5] and chimpanzee [12] chromosome 16. Indeed, several studies have shown that reticulate evolutionary processes, such as nonallelic homologous recombination (NAHR) and gene conversion occur within specific duplicon families, and molecular clock analysis and calculations based on sequence comparisons are confounded by these processes [40,44-46]. Interestingly, there are several reports presenting evidence that mutations in human *PKD1 *are caused via pseudogene-mediated gene conversion leading to ADPKD [37,39], [48]. Our own statistical analyses provided clear indications for gene conversion and/or NAHR that may have occurred among *PKD1 *family members in human and chimpanzee, respectively. One further aspect to be considered is the use of intron 30 for our sequence analysis and phylogenetic tree building. Our decision to focus on intron 30 was directed by the fact that the *PKD1 *exons are densely packed on the genomic level and intron 30 is the largest intron (> 1.5 kb) present in all *PKD1 *loci in human and chimpanzee, respectively. On the other hand, the evolutionarily highly conserved last intron (intron 45) of *PKD1 *[36] raises the question of whether there are other forces affecting intron 30 evolution. However, we did not find evidence of high sequence conservation in intron 30.

In conclusion, notwithstanding our results using intron 30 for phylogenetic tree building, it seems more likely that all six *PKD1*-pseudogenes evolved in a common ancestor of human and chimpanzee. After separation of both lineages there was internal correction of the genes/pseudogenes resulting in co-evolution of these genes within each species. By inference from an equally complex FISH-signal pattern of *PKD1 *shown in gorilla 16p, the species gorilla may well be included in this scenario of *PKD1 *evolution, suggesting that the original duplication of *PKD1 *may have occurred before gorillas and humans diverged ~8MYA.

## Abbreviations

ADPKD: autosomal dominant polycystic kidney disease; BAC: bacterial artificial chromosome; DAPI: 4',6-diamidino-2-phenylindole; FISH: Fluorescence *in situ *hybridization; ISCN: International system for human cytogenetic nomenclature; MYA: million years ago; NAHR: non-allelic homologous recombination; *PKD1*: polycystic kidney disease 1.

## Authors' contributions

SK performed parts of the molecular phylogenetic analyses and helped to finalize the manuscript, JP and CM performed the FISH experiments, AW and MK performed the statistical analyses and helped to finalize the corresponding sections of the manuscript, NB and PP performed probe identification, preparation and characterization, BD and WS designed the study, and WS drafted and finalized the manuscript.

## Supplementary Material

Additional file 1 **Phylogenetic tree of *PKD1 *intron 30 sequences.** Phylogenetic tree using sequences orthologous and paralogous to human intron 30 from human, chimpanzee, orangutan, and rhesus macaque. Human branch termini are labeled either by the functional gene or its pseudogene copies according to the nomenclature of NCBI Build 36. Chimpanzee branch termini are either labeled by the functional gene or with a pseudogene number (P1-P6) correlated to the Acc.No. of the corresponding contig in the PanTro 2.1 reference assembly (see Results section for detail). Bootstrap values are indicated on the respective branch. Click here for file

Additional file 2 **Pairwise sequence identities among all gene/pseudogene intron 30 sequences.** Intron 30 sequences of all gene/pseudogene copies are aligned by CLUSTALW, gap-containing positions removed and pairwise nucleotide sequence identities directly calculated from the CLUSTALW alignments. Click here for file

Additional file 3 **GENECONV analysis of human and chimpanzee *PKD1 *intron 30 sequences.** The first column lists the two sequences in which a significant fragment in the alignment was identified. Both p-values are multiple-comparison corrected for all sequence pairs, as well as for the length of the alignment. The SIM p-value indicates the likelihood to which such a similarity could be observed by chance. The BC KA p-value indicates the more conservative Bonferroni-corrected Karlin-Altschul value. The precise position of the identified recombinant sequence within the alignment is given in the fourth column. The final three columns summarize the number of polymorphic sites, the overall number of different sites and the mismatches between the two sequences compared. Click here for file
