# Chromosomal evolution of the *PKD1 *gene family in primates

**DOI:** 10.1186/1471-2148-9-14

**Published:** 2009-01-16

**Authors:** Stefan Kirsch, Juanjo Pasantes, Andreas Wolf, Nadia Bogdanova, Claudia Münch, Arseni Markoff, Petra Pennekamp, Michael Krawczak, Bernd Dworniczak, Werner Schempp

**Affiliations:** 1 Institut für Humangenetik und Anthropologie, Universität Freiburg, Breisacher Str. 33, 79106 Freiburg, Germany; 2 Institut für Medizinische Informatik und Statistik, Universität Kiel, Brunswiker Str. 10, 24105 Kiel, Germany; 3 Institut für Humangenetik, Universität Münster, Vesaliusweg 12-14, 48129 Münster, Germany; 4 Department of Biochemistry, Genetics and Immunology, University of Vigo, E-36200 Vigo, Spain; 5 Institut für Medizinische Biochemie, Universität Münster, Von Esmarch Str. 56, 48149 Münster, Germany

## Abstract

Correction to Kirsch S, Pasantes J, Wolf A, Bogdanova N, Münch C, Pennekamp P, Krawczak M, Dworniczak B, Schempp W: **Chromosomal evolution of the *PKD1 *gene family in primates**. BMC Evolutionary Biology 2008, **8**:263 (doi:10.1186/1471-2148-8-263)

## Correction

After publication of this work [1], we noted that we inadvertently failed to include the complete list of all co-authors. The full list of authors including Arseni Markoff has now been added.

## Authors

Stefan Kirsch, Juanjo Pasantes, Andreas Wolf, Nadia Bogdanova, Claudia Münch, Arseni Markoff, Petra Pennekamp, Michael Krawczak, Bernd Dworniczak and Werner Schempp

## Authors' contributions

SK performed parts of the molecular phylogenetic analyses and helped to finalize the manuscript, JP and CM performed the FISH experiments, AW and MK performed the statistical analyses and helped to finalize the corresponding sections of the manuscript, NB, AM and PP performed probe identification, preparation and characterization, BD and WS designed the study, and WS drafted and finalized the manuscript.
