# Supplementary figures and images for: Chromosomal evolution of the PKD1 gene family in primates
Source: BMC Evol Biol. 2008 Sep 26;8:263. doi: 10.1186/1471-2148-8-263 (PMC2564946; doi:10.1186/1471-2148-8-263)

**Additional File 1:**

**Phylogenetic tree of PKD1 intron 30 sequences**

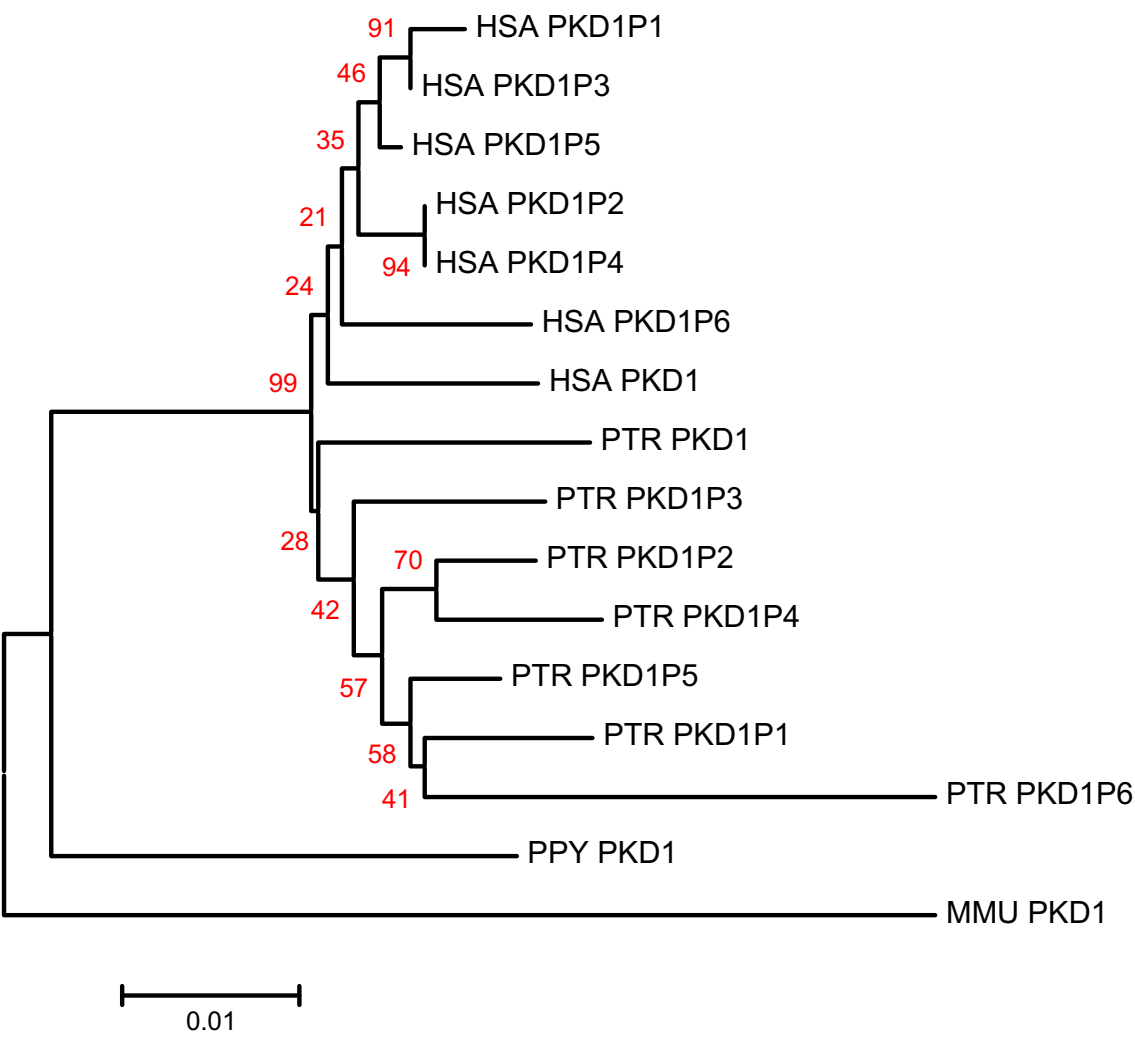

Supplement: Additional file 1 — Phylogenetic tree of PKD1 intron 30 sequences. Phylogenetic tree using sequences orthologous and paralogous to human intron 30 from human, chimpanzee, orangutan, and rhesus macaque. Human branch termini are labeled either by the functional gene or its pseudogene copies according to the nomenclature of NCBI Build 36. Chimpanzee branch termini are either labeled by the functional gene or with a pseudogene number (P1-P6) correlated to the Acc.No. of the corresponding contig in the PanTro 2.1 reference assembly (see Results section for detail). Bootstrap values are indicated on the respective branch. [file 1471-2148-8-263-S1.pdf]
