# Supplementary material for: Chromosomal evolution of the PKD1 gene family in primates
Source: BMC Evol Biol. 2008 Sep 26;8:263. doi: 10.1186/1471-2148-8-263 (PMC2564946; doi:10.1186/1471-2148-8-263)
Supplement: Additional file 2 — Pairwise sequence identities among all gene/pseudogene intron 30 sequences. Intron 30 sequences of all gene/pseudogene copies are aligned by CLUSTALW, gap-containing positions removed and pairwise nucleotide sequence identities directly calculated from the CLUSTALW alignments. [file 1471-2148-8-263-S2.pdf]

Additional File 3:

Pairwise sequence identities among all gene/pseudogene intron 30 sequences

	HSA PKD1	HSA PKD1P1	HSA PKD1P5	HSA PKD1P3	HSA PKD1P2	HSA PKD1P4	HSA PKD1P6	PTR PKD1P2	PTR PKD1P1	PTR PKD1P3	PTR PKD1P4	PPY PKD1	PTR PKD1P5	PTR PKD1P6	MMU PKD1	PTR PKD1
HSAPKD1	ID															
HSAPKD1P1	0,980	ID														
HSAPKD1P5	0,984	0,995	ID													
HSAPKD1P3	0,980	0,995	0,995	ID												
HSAPKD1P2	0,984	0,989	0,993	0,991	ID											
HSAPKD1P4	0,984	0,989	0,993	0,991	1,000	ID										
HSAPKD1P6	0,979	0,982	0,986	0,982	0,987	0,987	ID									
PTRPKD1P2	0,974	0,978	0,980	0,978	0,982	0,982	0,976	ID								
PTRPKD1P1	0,978	0,981	0,983	0,981	0,983	0,983	0,978	0,984	ID							
PTRPKD1P3	0,978	0,979	0,981	0,979	0,981	0,981	0,976	0,981	0,981	ID						
PTRPKD1P4	0,971	0,974	0,977	0,974	0,979	0,979	0,974	0,986	0,979	0,976	ID					
PPYPKD1	0,947	0,953	0,953	0,953	0,953	0,953	0,948	0,948	0,953	0,948	0,945	ID				
PTRPKD1P5	0,972	0,978	0,980	0,978	0,978	0,978	0,974	0,979	0,982	0,976	0,978	0,943	ID			
PTRPKD1P6	0,953	0,956	0,958	0,956	0,958	0,958	0,953	0,960	0,964	0,957	0,954	0,928	0,959	ID		
MMUPKD1	0,914	0,920	0,920	0,920	0,920	0,920	0,917	0,919	0,921	0,915	0,916	0,915	0,913	0,899	ID	
PTRPKD1	0,977	0,978	0,980	0,978	0,979	0,979	0,974	0,977	0,976	0,980	0,971	0,947	0,970	0,953	0,914	ID
