# Supplementary material for: Chromosomal evolution of the PKD1 gene family in primates
Source: BMC Evol Biol. 2008 Sep 26;8:263. doi: 10.1186/1471-2148-8-263 (PMC2564946; doi:10.1186/1471-2148-8-263)
Supplement: Additional file 3 — GENECONV analysis of human and chimpanzee PKD1 intron 30 sequences. The first column lists the two sequences in which a significant fragment in the alignment was identified. Both p-values are multiple-comparison corrected for all sequence pairs, as well as for the length of the alignment. The SIM p-value indicates the likelihood to which such a similarity could be observed by chance. The BC KA p-value indicates the more conservative Bonferroni-corrected Karlin-Altschul value. The precise position of the identified recombinant sequence within the alignment is given in the fourth column. The final three columns summarize the number of polymorphic sites, the overall number of different sites and the mismatches between the two sequences compared. [file 1471-2148-8-263-S3.pdf]

ADDITIONAL FILE 2:

GENECONV analysis of human gene/pseudogene intron 30 sequences

Similar sequences	Sim p-value	BC KA p-value	Aligned offsets Begin	End	No. of poly-morphic sites	No. of different sites	Mis-matches
HSAPKD1P3 : HSAPKD1P2	0.0068	0.09145	1	637	21	18	None
HSAPKD1P1 : HSAPKD1P4	0.0072	0.09257	1	522	15	24	None
HSAPKD1P3 : HSAPKD1P4	0.0106	0.12265	1	613	18	20	None
HSAPKD1P1 : HSAPKD1P2	0.0231	0.21611	1	522	15	22	None

GENECONV analysis of chimpanzee gene/pseudogene intron 30 sequences

Similar sequences	Sim p-value	BC KA p-value	Aligned offsets Begin	End	No. of poly-morphic sites	No. of different sites	Mis-matches
PTRPKD1 : PTRPKD1P2	0.0295	0.17346	33	221	23	25	None
